# Pilot randomised trial of a brief intervention for comorbid substance misuse in psychiatric in‐patient settings

**DOI:** 10.1111/acps.12530

**Published:** 2015-11-21

**Authors:** H. L. Graham, A. Copello, E. Griffith, N. Freemantle, P. McCrone, L. Clarke, K. Walsh, C. A. Stefanidou, A. Rana, M. Birchwood

**Affiliations:** ^1^ School of PsychologyUniversity of BirminghamEdgbastonBirminghamUK; ^2^ School of PsychologyUniversity of BathClaverton DownBathUK; ^3^ Department of Primary Care and Population HealthUpper Third FloorUCL Medical School (Royal Free Campus)LondonUK; ^4^ Health Service and Population Research DepartmentInstitute of PsychiatryKings College LondonLondonUK; ^5^ Division of Mental Health and WellbeingUniversity of WarwickCoventryUK

**Keywords:** randomised controlled trial, hospital admission, dual diagnosis, schizophrenia, substance misuse

## Abstract

**Objective:**

This proof of principle study evaluated the effectiveness and feasibility of a brief motivational intervention, delivered in mental health in‐patient settings, to improve engagement in treatment for drug and alcohol misuse.

**Method:**

A randomised controlled trial using concealed randomisation, blind, independent assessment of outcome at 3 months. Participants were 59 new adult admissions, to six acute mental health hospital units in one UK mental health service, with schizophrenia related or bipolar disorder diagnoses, users of community mental health services and also misusing alcohol and/or drugs. Participants were randomised to *Brief Integrated Motivational Intervention* (BIMI) with *Treatment As Usual* (TAU), or TAU alone. The BIMI took place over a 2‐week period and encouraged participants to explore substance use and its impact on mental health.

**Results:**

Fifty‐nine in‐patients (BIMI *n* = 30; TAU *n* = 29) were randomised, the BIMI was associated with a 63% relative odds increase in the primary outcome engagement in treatment [OR 1.63 (95% CI 1.01–2.65; *P* = 0.047)], at 3 months. Qualitative interviews with staff and participants indicated that the BIMI was both feasible and acceptable.

**Conclusion:**

Mental health hospital admissions present an opportunity for brief motivational interventions focussed on substance misuse and can lead to improvements in engagement.

**Significant Outcomes**

- It is both feasible and acceptable for in‐patient staff to engage in‐patient's in brief motivational interventions focussed on their substance misuse, whilst admitted to psychiatric hospitals. Staff who delivered the intervention found the short burst and targeted style a useful method of engaging in‐patients.
- The findings indicate that the brief motivational intervention, delivered during acute psychiatric admission, led to improvements in engagement in substance misuse treatment.
- Whilst this is a challenging group of participants to engage, it is clear that it is possible to recruit and retain participants from this client group and primary outcome data were available for 58, highlighting the advantage of a primary outcome measure based on assessment by care coordinators.

**Limitations**

- This was a pilot feasibility study, which supports the need for a larger trial.
- Specialist ‘dual diagnosis’ staff delivering the intervention alongside in‐patient staff provided the latter with an additional level of available expertise, informal *in‐situ* supervision and formed a bridge with care coordinators in the community. However, the availability of such a resource is limited in routine services and adds organisational complexity.
- Further research will need to test whether the intervention impacts significantly to reduce subsequent hospital admissions and substance misuse.

## Introduction

Hospital admissions may present a natural window of opportunity for in‐patients to re‐evaluate behaviours that might negatively impact on their health and mental health 1, 2, 3. As acute symptoms of mental ill health decline this period may be characterised as a time of contemplation and a window of increased awareness and insight into factors that contributed to becoming mentally unwell and or being admitted into hospital 4, 5. Research has shown that, some individuals may ‘seal over’ the experience, in an attempt to reduce emotional distress 6. That is, they may deny or minimise recent mental health symptoms or experiences and precipitating factors, and as a result, lose awareness of the triggers for becoming unwell 6. *Sealing over* the experience of relapse was found to predict low engagement with mental health services 6 months after discharge for psychiatric in‐patients 6. Drug and alcohol misuse in those with severe mental health problems is widespread 7, 8, 9, 10. It is associated with poor engagement in treatment 11, 12, 13, low motivation to change 14, 15, 16, increased psychiatric hospital admissions 17 and impacts negatively on the delivery of treatment and management of care during in‐patient stays 18. Among this client group, poor treatment engagement is a barrier for change and positive treatment outcomes 12, 16, 19, 20.

Of those admitted into psychiatric in‐patient facilities 22–44% have been found to have coexisting alcohol or drug problems 18. However, drug or alcohol problems and the role they may have played in precipitating a psychiatric hospital admission are not routinely addressed in such settings. It is recommended that the clinical management strategy of a psychiatric admission should involve in‐patient staff who are trained in routine assessment and treatment of substance misuse providing simple approaches to enhance motivation to change substance use 18. Nonetheless, this continues to be a significant gap in service provision, re‐admissions are high, outcomes for this group remain poor 21, 22. There are equivocal findings in trials of effectiveness of long‐term interventions with psychosis and substance misuse 23. However, the evidence for brief motivational‐based interventions is encouraging 16, 24, 25, 26, 27, 28 and shows promise in in‐patient settings when delivered by trained therapists 26, 29, 30.

### Aims of the study

The current study sought to evaluate whether a psychiatric hospital admission represents a natural window of opportunity for individuals who misuse substances to be routinely offered treatment, by in‐patient staff, to re‐evaluate drug and alcohol use and become aware of negative impacts on mental health.

## Material and methods

### Study design and participants

The study was an open (rater blinded), prospective randomised trial, analysed by intention to treat 31. Intention to treat according to ICH E9 is the principle that participants are analysed on the basis of the group to which they were randomised regardless of the treatment that they actually received. The trial utilised concealed randomisation; blind, independent assessment of outcome at 3 months; characterisation of refusers and drop‐outs. Participants were randomised on a 1:1 basis, to one of two experimental conditions: Brief Motivational Intervention (BIMI) in addition to Treatment As Usual (TAU); or TAU. Participants recruited were as follows: adults aged 18 years or above with an ICD‐10 diagnosis of schizophrenia, schizoaffective or delusional disorders (F20, 22, 23, 25, 28, 29); bipolar affective disorders (F31); or recurrent depressive disorder (F33.2) 32, service users of community mental health services; new admissions within the acute phase of severe mental health problems; who were identified as misusing alcohol and/or drugs over the past month based on a minimum score of 3 (abuse/dependent use based on DSM‐IV diagnostic criteria for substance‐related disorders) on the Clinicians Alcohol/Drugs Use rating scale (CDUS/CAUS) over the past 3 months 32 and had a care coordinator in a Community Mental Health Team. Participant's capacity to consent was established by the Responsible Clinical Officer. Participants were recruited from in‐patient units, within a single UK, National Health Service (NHS) Trust including eleven acute wards and three Psychiatric Intensive Care Units (PICUs), offering a total of 202 beds over a 15‐month period. The trial received ethical approval from the West Midlands – The Black Country National Research Ethics Committee (12WM/0369).

The primary hypothesis tested was that *Brief Integrated Motivational Intervention* (BIMI) would significantly improve treatment engagement for alcohol and drug misuse compared to TAU. The secondary hypotheses were that those receiving the BIMI would show greater readiness to change substance use behaviour when compared to those receiving TAU and that the BIMI would be more cost‐effective than the TAU due to reduced service utilisation.

Eligible participants were identified by Research staff based on review of care records. A screening measure was completed with care coordinators confirming trial eligibility. Within 2 weeks of admission, once acute symptoms eased, eligible participants were invited to participate. Written information about the study was provided and written consent obtained. A battery of assessments was administered. Upon completion, participants were randomly allocated to the intervention BIMI group (in the context of TAU) or the TAU group (the control group). Just prior to the 3‐month data collection meetings were scheduled for completion of follow‐up assessment battery by blind researchers. Participants were not paid for study participation.

### Sample size

A power calculation was carried out based on a previous study 27 using the primary outcome measure: Substance Abuse Treatment scale (SATs). Allocating 68 participants by a 1:1 strategy between the treatment and control conditions (34 participants per group) would have 90% power (1‐beta) to find a difference of 1 point on the SATs scale to be statistically significant, using a conventional two‐sided alpha of 0.05. A 1‐point difference would be clinically important for participants and mental health services as it would indicate increasing levels of engagement in treatment and addressing substance misuse (e.g. a shift from ‘Pre‐engagement’ to ‘Engagement’).

### Randomisation

The trial used independent central randomisation using a concealed process via e‐mail. The researchers were blind to participant treatment group allocation until all baseline, post‐treatment and 3‐month follow‐up quantitative assessments had been completed. Researchers were unblinded once the 3‐month data collection had been completed. Participants in the BIMI group then completed a qualitative interview.

### Trial Interventions

#### Brief integrated motivational intervention

The BIMI, a *‘Brief Integrated Motivational Intervention’*, was offered in the context of TAU, guided by a manual designed for purpose, based on key ingredients in the two early phases of Cognitive‐Behavioural Integrated Treatment 33 strategies from Cognitive therapy for substance use 34 and Motivational approaches 35. BIMI promoted a conversational style to build good collaborative relationships with participants working towards a joint goal of ‘keeping participants from returning to hospital’. The BIMI provided a 3‐step framework. The initial step provided participants with personalised feedback of information gathered in the baseline substance use assessment and highlighted potential impacts on health and mental health. Participants were provided with tailored psychoeducational material. The second step aimed to help participants make decisions. Strategies were aimed at: increasing awareness of perceived ‘benefits’ of use and ‘costs’ associated with continued misuse; re‐evaluation of positive beliefs about substances; building awareness of how substance use and mental health interact and maintenance cycles. The third step encouraged participants to contemplate change and develop a change plan based on a self‐identified goal and included strategies to cope with setbacks, cravings and urges and develop social support for change. Participants were offered a Peer Mentor during the second step of the intervention aiming to provide ongoing support and solidarity for change.

The BIMI was delivered by in‐patient unit staff trained as part of the study working alongside staff from a specialist ‘dual diagnosis’ Trust‐wide service, the COMPASS Programme 36. The BIMI was delivered over a 2‐week period for 4–6 sessions lasting 15–30 min each. A ‘booster session’ was arranged to be delivered 1 month after completion. The booster session aimed to consolidate motivation and transfer the skills from the BIMI to the community and was provided by a member of the specialist team and attended by the participant's community‐based care coordinator. Staff training was delivered over 2 days and supported by a treatment manual. Supervision of the BIMI was provided by three of the Investigators. Group, face‐to‐face or telephone supervision was delivered. During supervision, the standard of delivery was regularly monitored and assessed for fidelity and adherence.

### Treatment as usual

Treatment as usual was provided by nursing and medical staff on the mental health units in line with in‐patient policies and is regularly monitored by the UK Care Quality Commission. It included assessment and monitoring mental state, provision of medication and stabilisation of mental state.

### Statistical methods

Analysis was carried out using sas version 9.4 (SAS Institute, Cary NC, USA). The analysis method for the primary outcome was a proportional odds model. The proportional odds method used included the pre‐ and postintervention periods (L1SATS and L3STATS), included a cumulative log link function and multinomial error, linked within a subject with a random intercept term. Denominator degrees of freedom were derived from the number of subjects. Models used maximum likelihood with adaptive quadrature. The appropriateness of the proportional odds assumption was assessed using the Score Test. Statistical significance was assessed using a conventional two‐sided alpha of 0.05. The secondary outcomes were analysed using analogous generalised mixed models with appropriate link functions and error structures. The ITT principle was used for all analyses. Missing data were excluded as it would be inappropriate to impute it because it is most plausibly missing not at random.

### Cost‐effectiveness evaluation

Participant contacts with staff providing the intervention were recorded as well as staff supervision time, and these were combined with unit costs 37. Other service use was measured for the 3 months prebaseline and over the postrandomisation period using the Client Service Receipt Inventory 38. This recorded use of primary and secondary health and social care services. Sources of data included Client self‐report and where data could not be obtained via self‐report and/or specific information was required (e.g. medication, appointments, dates of hospital admission) this was gathered via the service electronic medical records. Total service costs were compared at baseline and over the postrandomisation period, the latter adjusting for the baseline costs. The EQ‐5D 39 measure was used, which is frequently used to produce quality‐adjusted life years as part of economic evaluations 31. Cost data are frequently skewed, and a bootstrap model was used which makes no assumption about the underlying cost distribution.

### Qualitative evaluation

Qualitative semistructured interviews with participants after the 3‐month follow‐up point sought to assess satisfaction with the treatment received and perceived processes of change, including helpful aspects of the therapeutic process. A sample of therapists was interviewed using a semistructured interview, individually and in focus groups. These were recorded, transcribed and thematic analysis conducted, steps were taken to ensure reflexivity 40. That is, attending systematically to the context of knowledge construction by examining concepts critically and aiming to eliminate assumptions and preconceptions affecting interpretation of findings. This was carried out through group discussions between the analysts, with the wider research team and checking the final findings with some participants 40.

### Outcome measures

#### Primary outcome

The primary outcome was engagement with substance misuse treatment whilst in‐patient and with community treatment services at 3‐month follow‐up as reported by care coordinators/primary clinician, using the SATS 14, 32a widely used measure with reported high validity, reliability and test–retest reliability 14, 32. The Primary Clinicians assessed the level of engagement in substance misuse treatment and progress towards recovery from substance use problems, based primarily on observable behaviours, on an 8‐point hierarchical motivational scale. The reporting timeframe was adapted to the previous 3 months.

#### Secondary outcome measures

##### Readiness to change

Current readiness to change alcohol and drug use was assessed using the 19‐item *Stages of Change Readiness And Treatment Eagerness Scale* 41.

Motivation to change was measured by the *Importance‐Confidence Ruler* which is a global assessment of motivation and confidence to change assessing two concepts underpinning readiness to change 42; participants were also asked to rate on a scale of 0–10 how important it was to change the use of the primary substance and how confident they were that they would succeed.

##### Drug use and alcohol use

Drug and alcohol use was assessed using a number of complementary measures. The number of days each substance had been used in the past 30 days, and the average amount of use of each drug on a using day was assessed using section B of the *Maudsley Addiction Profile* 43. The CDUS/CAUS was used as a screening tool for inclusion, based on DSM‐IV diagnostic criteria for substance‐related disorders, and reliably used by primary Clinician's to classify severity of substance use among people with severe mental health problems 32. The reporting timeframe for the AUDIT both at baseline and post‐treatment was adapted to the previous 3 months. The *Severity of Dependence Scale* was used to screen for the severity of drug dependence 44, 45, 46. The *Alcohol Use Disorders Identification Test* (AUDIT) a well validated 10‐item self‐report questionnaire was used to investigate alcohol consumption 47, 48.

##### Psychological functioning

Psychological functioning was assessed using three measures: Recovery Style Questionnaire (RSQ) is a 39‐item self‐report measure designed to assess two concepts of recovery from mental health relapses; ‘Integration’ (i.e. acknowledgement of, openness and attempts to cope with the mental health problem), and ‘Sealing‐over’ (i.e. cognitive and behavioural attempts at avoiding the diagnosis and experience of mental health problems). It reliably enables four recovery styles to be classified based on a continuum [i.e. ‘Integration’; ‘mixed picture in which integration predominates’; ‘mixed picture in which sealing‐over predominates’ and ‘sealing‐over’^49^]; Insight Scale reliably assesses changes in levels of insight in terms of perceived need for treatment 50; HADS a 14‐item self‐report measure found to reliably assess anxiety and depression 51, 52.

## Results

**Figure 1:**
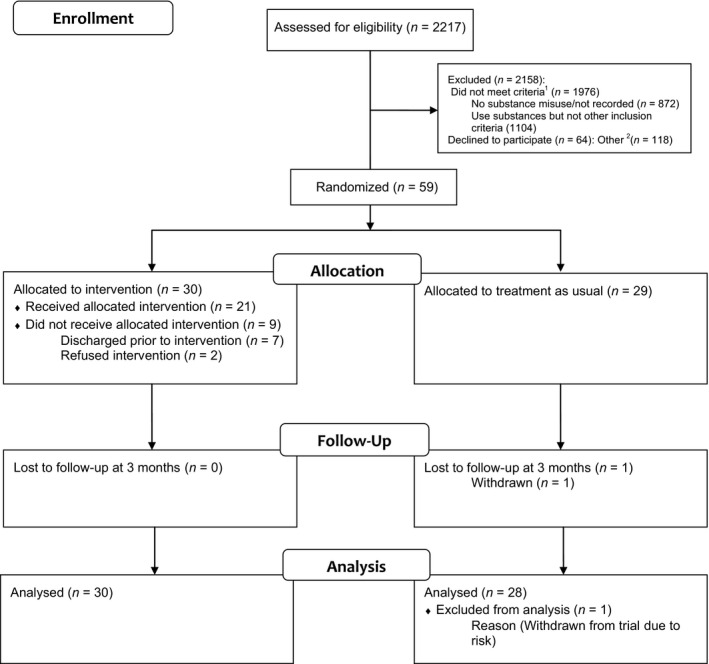
CONSORT diagram of trial profile. ^1^Did not meet inclusion criteria: No substance misuse/Substance use not recorded (*n* = 872), Use substances but not other inclusion criteria (*n* = 1104) (Inclusion criteria not met due to either, mental health, care co‐ordinator unavailable/unallocated, rating unavailable, discharged prior to rating, language, mental health capacity, care being transferred to or from another service). ^2^Other: includes previously consented (*n* = 6), previously refused (*n* = 15), previously withdrawn (*n* = 1), discharged before rating (*n* = 30), unable to rate (*n* = 28), discharged before met (*n* = 32), not randomized – withdrawn (*n* = 4), not randomized – discharged (*n* = 2).

A total of 60.67% (1345/2217) of in‐patients screened were using substances. However, 1286 did not meet the other study criteria or declined to be involved in the study. A total of 44%, (872/1976) of in‐patients were not misusing substances, or their substance use was not recorded (Fig. 1). Fifty‐nine in‐patients were recruited from 14 wards (Acute *n* = 50; PICU *n* = 9) and randomised to BIMI *n* = 30 or TAU *n* = 29. Table 1 shows baseline characteristics of the participants who had a mean age of 38.6 years, with a schizophrenia diagnosis, primarily misusing cannabis, or alcohol. In‐patients (*N* = 123) were approached regarding involvement in the study, the characteristics of those who consented compared to refusers (*n* = 64) (52%) appeared similar in age, sex and type of mental health team. All participants were followed up for the primary outcome at 3 months, one participant was withdrawn due to risks. Fifty (85%) study participants were interviewed at 3‐month follow‐up.

**Table 1:** Baseline characteristics of participants

	TAU (*n* = 29)	BIMI (*n* = 30)
Age		
Mean (SD)	37.69 (11.11)	39.5 (11.12)
Sex (%)		
Male	25 (86.2)	25 (83.3)
Diagnosis (%)		
Schizophrenia	19 (65.5)	17 (56.7)
Schizoaffective disorder	2 (6.9)	3 (10)
Psychosis	1 (3.4)	0 (0)
Bipolar affective disorder	7 (24.1)	10 (33.3)
Primary substance (%)		
Alcohol	11 (37.9)	12 (40)
Cannabis	13 (44.8)	14 (46.7)
Crack	0 (0)	1 (3.3)
Cocaine	2 (6.9)	0 (0)
Methadone	1 (3.4)	0 (0)
Legal Highs	2 (6.9)	2 (6.7)
Diamorphine	0 (0)	1 (3.3)
Ethnic origin (%)		
White British	15 (51.7)	7 (23.3)
White Irish	2 (6.9)	4 (13.3)
Asian Indian	2 (6.9)	0 (0)
Asian Pakistani	1 (3.4)	4 (13.3)
Asian Bangladeshi	1 (3.6)	0 (0)
Asian Other	1 (3.4)	1 (3.3)
Black Caribbean	4 (13.8)	8 (26.7)
Black African	2 (6.9)	0 (0)
Black Other	0 (0)	1 (3.3)
Mixed Caribbean	0 (0)	5 (16.7)
Mixed African	1 (3.4)	0 (0)
Housing status (%)		
Live alone	16 (55.2)	16 (53.3)
With parents or guardian	5 (17.2)	9 (30)
With partner	3 (10.3)	0 (0)
Other	5 (17.2)	5 (16.7)

### The intervention

Twenty seven in‐patient staff from all wards and six specialist staff were trained to deliver the BIMI, 12 in‐patient staff and five specialist staff actually delivered the MI. Twenty‐one of the 30 (70%) participants in the BIMI group received the intervention. The nine participants who did not were discharged prior to the first session (*n* = 5), refused the intervention (*n* = 3) and sent on home leave (*n* = 1). The mean exposure for participants who received the intervention was 3.14 (SD 1.92) sessions (range 1–5 sessions). Mean duration of sessions was 18.3 min (SD 4.90). The average total mean duration of the intervention was 57.6 min (SD 31.33). The booster session was completed for nine (30%) of these participants. Two participants received a session with a Peer Mentor who aimed to provide ongoing support and solidarity for change. The Peer Mentor provided some low‐key social support and a listening ear to help participants feel less isolated and more connected to others during a challenging time and to generally see how they were finding the intervention.

### Primary outcome

#### Engagement with substance misuse treatment

At baseline, for both groups engagement could be described as not having regular contact with an assigned case manager. Both groups had shifted at follow‐up, indicating regular contacts with a case manager and discussing substance use and showing evidence of a reduction in use. The effect of randomisation to the BIMI group rather than TAU was described using the odds ratio where a value greater than 1 indicates a benefit for the intervention. Two TAU SATs values were missing, one at baseline the other at follow‐up. The intervention was associated with a 63% relative odds increase in the SATs score, which is conventionally modestly statistically significant [OR 1.63 (95% CI 1.01–2.65; *P* = 0.047)] (Table 2). In supportive analyses, the relationship between actual length of stay in hospital and response to the intervention in relation the primary outcome was examined. There was no interaction between time in hospital and treatment effect.

**Table 2:** Difference in Substance Abuse Treatment scale (SATs), number of substances used, days using and HADS at 3‐month follow‐up by randomised condition, conditional on baseline assessment

Outcome	Odds Ratio^a^ (95% CI)	Relative Risk^b^ (95% CI)	Difference in means^c^ (95% CI)	*P* value^d^
SATs	1.63 (1.01–2.65)	–	–	0.047^e^
Number of substances used	0.66 (0.33–1.33)	–	–	0.240
Number of days using primary substance	–	1.02 (0.82–1.26)	–	0.854
HADS				
Anxiety	–	–	−0.80 (3.93–2.34)	0.611
Depression	–	–	−1.89 (−4.51 to 0.74)	0.156
Insight Scale				
Awareness of symptoms	–	–	0.03 (−0.70 to 0.75)	0.944
Awareness of illness	–	–	0.25 (−0.42 to 0.93)	0.459
Need for treatment	–	–	0.09 (−0.65 to 0.84)	0.802
Total	–	–	1.03 (−0.49 to 2.54)	0.178

a Mixed proportional odds model.

b Poisson mixed model

c Gaussian mixed model

d Two‐tailed.

e *P* < 0.05.

### Secondary outcome measures

#### Readiness to change

For the secondary outcome, readiness to change, both groups remained in the ‘low’ readiness to change category for alcohol and drugs at follow‐up. No overall statistical analysis was carried out for this readiness to change data, due to missing data and the scores for alcohol and drug use could not be meaningfully combined. Assessment of motivation to change indicated that both groups at baseline similarly rated the Importance to change their substance use [BIMI = 6.77(SD 3.23); TAU = 7.19(SD 3.58)] and Confidence to be able to change [BIMI = 8.12(SD 2.30); TAU = 7.50(SD 2.94)]. There was very little variation between Importance [BIMI = 7.08(SD 3.74); TAU = 6.89(SD 3.30)] and Confidence [BIMI = 8.15(SD 2.19); TAU = 8.02(SD 2.83)] scores across time and between groups.

#### Substance use

##### Number of days substances used

The number of days in the past 30 in which the primary substance consumed was available for 50 subjects at baseline and follow‐up. Both groups reduced number of days they used by more than half (Table 3). There was no evidence of an effect of randomised treatment on the number of days using the primary substance [Relative risk 1.02 (95% CI 0.82–1.26; *P* = 0.85)] (Table 2).

**Table 3:** Substance Use in past 30 days at baseline and follow‐up

	BIMI		TAU	
	Baseline (*n* = 30)	Follow‐up (*n* = 26)	Baseline (*n* = 29)	Follow‐up (*n* = 24)
Number of substances				
None	0	10	0	5
One	14	10	18	16
Two	12	6	7	3
Three	4	0	3	0
Four	0	0	1	0
Number of days used				
Mean (SD)	21.48 (11.00)	9.25 (10.82)	21.23 (9.68)	9.31 (11.86)

In supportive analysis, the amount of substance used was explored. At 3‐month follow‐up, 15 participants reported not having used any substances in the past 30 days; 10 of whom were in the BIMI group compared to 5 in TAU (Table 3). The number of substances used by the BIMI group reduced on average by 0.34 or about a third of a substance when compared to the TAU group (Table 2). However, the effect was not significant [OR 0.66 (95% CI 0.33–1.33; *P* = 0.24)].

##### Severity and impact of substance use

Mean baseline scores for the primary substance used for both groups, as rated by the care coordinator, were categorised as ‘abuse’ in the previous 3 months (Table 4). Mean AUDIT scores for the TAU and BIMI group met the ‘possible dependence’ category at baseline. At 3‐month follow‐up, these mean scores were lower and were within the ‘increasing risk category’. Mean SDS scores across time and groups were all above a score of 4 which suggested a continued ‘level of dependence’.

**Table 4:** Severity and impact of primary substance at baseline and 3‐month follow‐up

	TAU		BIMI	
	Baseline	Follow‐up	Baseline	Follow‐up
Alcohol (CAUS)	*N* = 11	*N* = 11	*N* = 12	*N* = 12
Mean (SD)	3.27 (0.65)	2.18 (.98)	3.42 (0.67)	2.25 (1.22)
Drugs (CDUS)	*N* = 18	*N* = 17	*N* = 18	*N* = 18
Mean (SD)	3.28 (0.46)	2.41 (1.06)	3.33 (0.49)	1.89 (0.90)
Alcohol Use (AUDIT)	*N* = 11	*N* = 11	*N* = 12	*N* = 9
Mean (SD)	20.00 (8.14)	13.09 (7.92)	22.00 (7.76)	15.11 (7.71)
Drug Use (SDS)	*N* = 18	*N* = 13	*N* = 18	*N* = 14
Mean (SD)	5.11 (4.93)	5.31 (3.68)	4.56 (4.23)	4.64 (4.18)

#### Psychological functioning

##### Anxiety and depression

Analysis of the difference in the HADS Anxiety and Depression Scores by randomised group, accounting for baseline values, indicated no evidence of a treatment effect on HADS Anxiety (Difference in means −0.80 [95% CI 3.93–2.34; *P* = 0.611)]. The HADS Depression, although not significant, was in line with a modest effect on that outcome suggesting that the TAU had higher depression scores (Difference in means −1.89 [95% CI −4.51 to 0.74; *P* = 0.156)] (Table 2).

##### Style of recovery from mental health problems

Baseline mean scores on the RSQ descriptively classified the TAU group as adopting a ‘mixed picture in which integration predominates’ style of recovery from mental health problems [66.54 (SD 15.28)], and the BIMI group as adopting an ‘integration’ style [71.14 (SD 13.37)]. At 3‐month follow‐up, both groups were classified as ‘integration’ (TAU = 70.74 (SD 70.74); BIMI = 71.15(SD 18.97), suggesting that both groups had an acknowledgement of and were making attempts to cope with their mental health problems. Due to the amount of missing data, no further analysis was conducted.

##### Insight in mental health problems

Awareness of symptoms and of illness mean scores indicated ‘poor insight’ over time in both TAU groups at baseline and follow‐up. Need for treatment mean scores increased over time for both groups suggesting ‘good insight’ into need for treatment. The difference in means implied a benefit for the intervention. Whilst there were no significant differences, all values pointed towards a benefit from the BIMI (Table 2).

### Cost‐effectiveness evaluation

For the secondary outcome of cost‐effectiveness, service use in the period prior to baseline was relatively similar between the two groups. All participants were in‐patients during the baseline period and all but one were also in‐patients during the postrandomisation period (one participant in the BIMI group went on home leave on the day of randomisation). Although we do not test for differences for individual services, it was observed that during the postrandomisation period there was a greater number of participants in contact with psychiatrists in the BIMI group (BIMI *n* = 19; TAU *n* = 13) and more participants in contact with assertive outreach teams (BIMI *n* = 7; TAU *n* = 1). At baseline, the number of days in hospital was similar between the groups (BIMI = 13.4 (SD 7.9); TAU=14.5 (SD 9.7). During the post‐randomisation period, in‐patient days were slightly higher in the BIMI group (BIMI = 45.3 (SD 35.1); TAU = 35.8 (SD 30.8). The mean cost of delivering the intervention was £72 (SD £66). The total mean cost of services used postrandomisation by the TAU and BIMI group was £15 698 (SD £12 632) and £18 651 (SD £15 580) respectively. Adjusting for baseline costs, the BIMI group had costs that were on average £3279 higher postrandomisation than the TAU group (95% CI, −£3933 to £10 876). One participant in the BIMI group was an outlier in number of days of in‐patient care during the postrandomisation period. When they were removed, the mean cost for the BIMI group was £16 825 (SD £12 159). The EQ5D‐5L scores were similar between the two groups.

### Qualitative evaluation

Two focus groups (*n* = 7) and five individual interviews were conducted with staff who delivered the BIMI. Twenty‐one participants who received the intervention were interviewed at follow‐up. Qualitative data revealed that participants and staff found the BIMI both feasible and acceptable when delivered as part of routine care on in‐patient wards. Whilst most participants reported on positive non‐specific factors of the intervention, such as ‘staff giving time’ and ‘going out of their way’, several participants identified that the intervention allowed them to recognise the amount of substances they were using, the pros and cons/effects of their use on them or their mental health, as well as developing new coping strategies and techniques. For in‐patient staff and the specialist practitioners, the targeted, motivational style of the intervention and the brevity of the sessions were found to be useful to engage patients in discussions about their substance use. There were mixed views about the timing of the intervention and how well it worked when in‐patients were acutely unwell; other issues included the practicalities of joint working between in‐patient and community‐based staff; and conflict with duties on the ward.

## Discussion

The intervention was associated with an improvement in engagement in treatment, the primary outcome, which was modestly statistically significant providing some support for the principal hypothesis in line with findings in out‐patient settings 27. During the postrandomisation period, there were greater rates of contacts with psychiatrists and assertive outreach teams among in‐patients who received the intervention. A shift was observed from irregular to regular contacts with care coordinators, discussing substance use. Both groups fell into the ‘low’ readiness to change category at follow‐up, and there was no significant difference in the reduction in the number of days on which the primary substance was used between groups. However, both groups reported a reduction by more than half in the number of days they used their primary substance over the past 30 days at 3‐month follow‐up. These changes in substance use noted in both groups are not an uncommon findings in studies evaluating motivational‐based interventions. A number of explanations have been offered including the natural fluctuations in coexisting mental health and substance misuse and the period before an admission being associated with greater amounts of substance use resulting in relative improvement that may not necessarily reflect longer term improvements; also it has been suggested that the baseline assessment of both groups may have a motivational effect 53, 54, 55. Some exploratory non‐significant findings that warrant investigation in a larger trial are reductions in the number of substances used. A study by Baker and colleagues carried out in in‐patient settings similarly found that BIMIs had the potential to have a modest, short‐term impact on poly‐drug use 56. Tobacco smoking was not assessed in the current study; however, this would be an important consideration within future research due to its prevalence and impacts in this population.

The study aimed to assess the feasibility and acceptability of delivering a brief integrated motivational intervention to in‐patients as part of routine care on the wards. The recruitment rate in the current study was 50%, which was expected among this group of service users who have been historically described as ‘difficult to engage’ 11, 12, 13, 14, 27. However, 85% of participants were retained in the study at follow‐up and 70% of participants engaged in the BIMI with a good level of exposure to it. In‐patients described the intervention as helpful enabling them to: have an opportunity to talk to in‐patient staff, recognise the amount of substances they were using and the effects of their use on them including their mental health. Developing new coping strategies and techniques was also helpful. This study indicates that it is feasible and acceptable to engage and retain in‐patients, with severe mental health problems from a diverse range of ethnic origins, who are poorly engaged in treatment and misusing a range of substances in a brief intervention for substance misuse, whilst they are on a psychiatric wards. There was improved awareness in both groups over time of the need for mental health treatment and a positive shift to a more integrating style of recovery from mental health problems which has been associated with improved engagement in treatment over time 6. The intervention was relatively low cost, and no significant differences were observed in costs between the two groups. The rate of substance use in the in‐patient sample was 61%. However, despite the reported rates prevalence of substance misuse among those with severe mental health problems in the literature and in the current study, a number of in‐patients in the current study were unable to be included for a number of reasons. Key factors appeared to be whether information about substance use was reported and how it was reported and the stringent study inclusion criteria needed due to the nature of a randomised controlled trial. These factors have important implications for clinical practice and a further trial. Some of the eligible in‐patients who refused to participate were poorly engaged with services and with their in‐patient care and treatment; some were reluctant or suspicious of engaging in a research study to discuss their substance use. Future research might also consider strategies to increase the participation rate among eligible in‐patients, including payment for participation and intervening earlier on during an admission.

Unlike previous studies 26, 29, 30, the current study utilised routine in‐patient staff to deliver the intervention. They reported that the short burst and targeted style of the intervention was a useful method of engaging in‐patients in discussions about drug and alcohol use. However, they felt that the timing (i.e. when such an intervention is delivered) was important due to the mental state of in‐patients on acute wards. Similarly participants felt that sometimes they did not have the ‘headspace’ and so the timing of the intervention appears important. The qualitative findings highlight that a shift in the clinical management strategy on wards would be needed for such brief interventions to become embedded into routine practice 57, 58. The literature on in‐patient psychiatric treatment has highlighted the need for *‘protected time’* for staff to deliver therapeutic interventions as a routine part of their in‐patient work 59, 60, 61.

Specialist ‘dual diagnosis’ staff delivering the intervention alongside routine in‐patient staff provided an additional level of available expertise and informal supervision *in‐situ*. However, the availability of such a specialist resource is limited and adds another layer of organisational complexity. Nonetheless, the recommendations from other studies for comorbid severe mental health and substance misuse problems are that brief interventions are best provided within the context of a comprehensive package of ongoing integrated treatment 27, 62, and the specialist staff were able to form a bridge with care coordinators in the community. In future trials, the BIMI may benefit from evaluation as part of a comprehensive package of integrated treatment so that positive changes in engagement can be built upon in community services postdischarge.

This proof of principle study attempted to test the BIMI, method and measures in an in‐patient setting, and there are aspects that would require refinement for further studies. There were recruitment challenges; the original protocol aimed for recruitment of 68 participants, in the event we recruited 59. Whilst a challenging group of participants to engage, it is clear that it is possible to recruit and retain participants from this client group and primary outcome data were available for 58 highlighting the advantage of a primary outcome based on an assessment by care coordinators. However, although these primary Clinicians would have been blind at the baseline and 2‐week ratings, potentially they were not at the 3‐month rating for those in the intervention arm. This is a limitation of the study that may have introduced bias. In addition, a rating of this nature provided by the Primary Clinician working with the client might be influenced by the therapeutic alliance. Measuring motivation to change raised a number of challenges, including the sensitivity of the measures to differentiate between those who are abstinent or have made changes in substance use compared to those who are precontemplators. The low uptake of Peer mentors requires further consideration in future research. The low number of female in‐patients included in the study may well reflect that coexisting severe mental health, and substance misuse is more common among men and less prevalent among females. It is also questionable whether an improvement in costs and health‐related quality of life over a 3‐month time period would be found. A future trial should include a sufficiently long follow‐up to explore cost changes over a prolonged period. In particular, long enough to detect differences in readmission rates and assess changes in substance misuse. However, this pilot feasibility study suggests that an acute psychiatric hospital admission may present a ‘teachable moment’ similar to that identified in physical health settings 1, 63; that is a natural window of opportunity for in‐patient staff to routinely engage in‐patients in BIMIs focussed on alcohol and drug use. The intervention is feasible and represents a simple, low cost and easy to implement approach, however, timing is key.

## Declaration of interests

The authors’ declare that they have no competing interests. All authors have completed the Unified Competing Interest form (available on request from the corresponding author) and declare that (i) [HG, AC, MB, EG, NF, PMc, LC, KW, CS and AR] have support for the submitted work; (ii) [HG, AC, MB, EG, NF, PMc, LC, KW, CS and AR] have no relationships with any companies that might have an interest in the submitted work in the previous 3 years; (iii) their spouses, partners or children have no financial relationships that may be relevant to the submitted work; and (iv) [HG, AC, MB, EG, NF, PMc, LC, KW, CS and AR] have no non‐financial interests that may be relevant to the submitted work. The statistical analyses were performed by Professor Nick Freemantle Department of Primary Care and Population Health, UCL Medical School (Nicholas.Freemantle@ucl.ac.uk) and Professor Paul McCrone Health Service and Population Research Department, Institute of Psychiatry, Kings College London (paul.mccrone@kcl.ac.uk). Trial Registration: NIHR CRN/UKCRN:13978 date of registration – 06.02.13; ISRCTN43548483 date of registration – 4/17/2014.
